# Germ Line Origin and Somatic Mutations Determine the Target Tissues in Systemic AL-Amyloidosis

**DOI:** 10.1371/journal.pone.0000981

**Published:** 2007-10-03

**Authors:** Stina Enqvist, Knut Sletten, Fred J. Stevens, Ulf Hellman, Per Westermark

**Affiliations:** 1 Department of Genetics and Pathology, Uppsala University, Uppsala, Sweden; 2 Biotechnology Centre of Oslo, University of Oslo, Oslo, Norway; 3 Biosciences Division, Argonne National Laboratory, Argonne, Illinois, United States of America; 4 Ludwig Institute for Cancer Research, Uppsala, Sweden; Massachusetts Institute of Technology, United States of America

## Abstract

**Background:**

Amyloid is insoluble aggregated proteins deposited in the extra cellular space. About 25 different proteins are known to form amyloid *in vivo* and are associated with severe diseases such as Alzheimeŕs disease, prion diseases and type-2 diabetes. Light chain (AL) -amyloidosis is unique among amyloid diseases in that the fibril protein, a monoclonal immunoglobulin light chain, varies between individuals and that no two AL-proteins with identical primary structures have been described to date. The variability in tissue distribution of amyloid deposits is considerably larger in systemic AL-amyloidosis than in any other form of amyloidosis. The reason for this variation is believed to be based on the differences in properties of the amyloidogenic immunoglobulin light chain. However, there is presently no known relationship between the structure of an AL-protein and tissue distribution.

**Methodology/Principal Findings:**

We compared the pattern of amyloid deposition in four individuals with amyloid protein derived from variable light chain gene O18-O8, the source of a high proportion of amyloidogenic light chains, and in whom all or most of the fibril protein had been determined by amino acid sequencing. In spite of great similarities between the structures of the proteins, there was a pronounced variability in deposition pattern. We also compared the tissue distribution in these four individuals with that of four other patients with AL-amyloid derived from the L2-L16 gene. Although the interindividual variations were pronounced, liver and kidney involvement was much more evident in the latter four.

**Conclusions/Significance:**

We conclude that although the use of a specific gene influences the tissue distribution of amyloid, each light chain exhibits one or more determinants of organ-specificity, which originate from somatic mutations and post-translational modifications. Eventual identification of such determinants could lead to improved treatment of patients with AL amyloidosis.

## Introduction

Amyloid is insoluble fibrillar protein deposited in the extracellular space. The resulting heterogeneous group of disorders is called amyloidosis. The protein in the fibrils are hydrogen bound intermolecularly in β-sheets giving the fibrils a considerable strength and the structure explains most of the characteristic properties of amyloid. Today, about 25 proteins are known to form amyloid *in vivo* and these are involved in life-threatening disorders like type-2 diabetes and Creutzfeldt Jacob́s disease [1].

The systemic amyloidoses are characterized by widely spread deposits of specific proteins in a characteristic fibrillar form. Usually vital organs are affected and the disorders are therefore often lethal. More than ten different proteins are known to be able to give rise to systemic amyloidosis, most of them in rare, hereditary forms [1]. In systemic AL-amyloidosis, the amyloid fibril protein is derived from monoclonal immunoglobulin light chains. All light chain subtypes are capable of fibril formation although lambda chains, particularly of lambda-VI type, are over-represented.

The immunoglobulin light chain in systemic AL-amyloidosis is produced by plasma cells, mainly in the bone marrow. The protein is then circulating in plasma before it aggregates into the typical amyloid fibrils at sites far from the origin. Most commonly it is not the whole protein that is included but the variable region with a part of the constant region. Whether or not this C-terminal cleavage occurs before the assembly and is important in this process or is a result of proteolytic trimming of a part not included in the fibril core is still not known. Usually, full-length light chains are included in the fibril as a minor component [2]. On rare occasions, the major fibril protein is derived from the constant region [3]–[5].

Systemic AL-amyloidosis is probably the most heterogeneous form of amyloidosis that occurs. There is no general rule regarding where deposits may occur, which is different from most other systemic amyloidoses. Therefore, the clinical presentation is highly variable. In some patients, cardiac symptoms predominate, in others nephropathy. Again others present with polyneuropathy, gastrointestinal disturbances, liver affection and so on [6]. In accordance with the systemic nature of the disease, combinations are common and in reality, most organs are involved also when not giving symptoms. However, amyloid does not occur in the central nervous system [7].

It has been difficult to explain why only some individuals with production of monoclonal immunoglobulin light chains develop systemic amyloidosis. After an increasing number of amino acid sequences of AL-proteins have been obtained, it has become more clear that some unusual amino acid substitutions or even light chain subgroups [8] are over-represented in these proteins. In addition, glycosylation may be linked to AL amyloidogenesis [9]. Further studies have given an increasing body of information on the putative importance of certain amino acid substitutions in the AL-protein sequences [10]. Thus, it was found that over 80% of amyloidogenic κI variable light chains regions were characterized by the presence of at least one of three single-site amino acid substitutions or formation of an N-linked glycosylation site [10], [11]. Consequently, it is increasingly clear that the most important risk factor for the development of AL-amyloidosis is related to the structure (and concentration) of the monoclonal immunoglobulin light chain. Interestingly, at least for κ chains, these risk factors seem to be a random matter, since they may be identified in the normal antibody repertoire [12]–[14]. Amyloidosis may then be a consequence of a clonal expansion of a plasma cell producing these specific light chains.

The reasons for the variability in organ distribution in systemic AL-amyloidosis are not known. It is reasonable to believe that variations are due to properties related to differences in protein sequences. In addition to differences in properties in the individual light chains, it has been suggested that there is a preferential organ tropism for different variable light chain gene products [15], [16]. Thus, AL λVI is significantly more often associated with clinically important renal amyloidosis than other subtypes [15]. However, the literature is sparse concerning the nature of the amyloid light chain subgroup and distribution of amyloid in the body. This is in a high degree true for amyloid of AL κ type [16] although κI light chains are particularly well represented in immunoglobulin light chain sequences [14]. Moreover, performed studies are based on clinical evaluation of effects of amyloid on the organ in whole. Almost no studies on the structure of AL-proteins include any detailed histological analyses, allowing comparisons on how deposits are distributed in specific tissue structures. Finally, studies in which both the AL-protein sequence and the tissue distribution have been determined do not exist.

In the present study we have chosen to study four AL amyloid proteins of κIb type (O18-O8) and, for comparison, four amyloidogenic light chains of L2-L16 origin (κ3a). We have evaluated the distribution of amyloid deposits in the body from available tissues, obtained at autopsy. The use of the material and the study has been approved by the Ethical Committee of the Uppsala University Hospital.

## Results

### Comparison of the four AL-proteins of κ1b type

**Figure 1:**
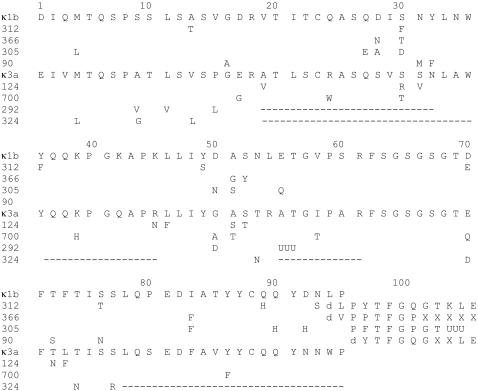
Primary structure of the κ1b and the κ3a light chains compared with respective germline gene sequence. ---- No sequence : d, deletion, UUU, end of the determined amino acid sequence.

The amino acid sequences of AL proteins Es305 and 366 have been reported [17]. For details concerning amino acid sequencing of AL 312 and AL 90, see supporting information (Text S1). The four proteins belong to the κIb immunoglobulin light chain subgroup and are derived from the O18-O8 gene, (Fig. 1). Most amino acid differences are uncommon or unique. None of the four kappa-1 light chains discussed here exhibit any of the three point mutations that are highly correlated with amyloidosis among kappa-1 light chains [14], i.e., Ile is found at position 29, Asp does not appear at position 31, and the salt-bridge involving Arg61 and Asp82 is intact in all of the proteins. On the other hand, two of the light chain proteins, Es 305 and AL 366, [17], incorporate the fourth “risk factor,” a glycosylation site.

The variable domains of these light chains have acidic pI values. Es305 at 5.4 is the most basic, with 366, 312, and 90 pI values computed at 4.1, 4.7, and 4.4 respectively. None of the amino acid replacements are over-represented in amyloidogenic kappa light chains. However, Phe36 (AL 312) has been seen in 27 other κ chains, of which 16 were known to be associated with amyloidosis [10].

Each of the four kappa-1 sequences exhibits one unique amino acid replacement: Thr13 (AL312), Met31 (AL90), Asn50 (Es305), and Val95 (AL366) [10]. In addition, AL312 and AL366 have a deletion at position 94. The rarity of these residue changes could suggest that these are structurally unfavorable alterations and that most kappa light chains that incorporate them do not become a component of functional antibodies, a prerequisite for eventual production by a neoplastic plasma cell. The uniqueness of Met31 and Val95 can also be explained by the fact that each of these mutations requires two base changes. The remaining amino acid replacements can be accomplished with single base changes.

Alternative amino acids that are observed at position 13 in Framework 1 are hydrophobic, the presence of the hydroxyl group in the Thr side chain (AL 312) may disrupt the hydrophobic core of the beta domain. Position 50 is a highly promiscuous site in CDR2, conceivably Asn50 (Es305) is highly destabilizing since all other charged and neutral amino acids are observed among kappa light chains. In such a case, the establishment of Es305 in a viable antibody, eventually leading to the production of a neoplastic plasma cell, could be the result of another somatic mutation in Framework 1, i.e., the fortuitous occurrence of a stabilizing Leu for Met replacement at position 4.

### Comparison of the AL-proteins of κ3a type

The four κ3a AL-proteins were included in this study mainly for comparison the deposition patterns between proteins of κ1b and κ3a type. Since full amino acid sequences could not be obtained for AL292 and AL324, no detailed molecular analysis was performed. We anticipate that destabilizing amino acid replacements contribute to the development of amyloid fibrils by the κ3a -proteins studied here. Unsurprisingly, most of the amino acid changes were innocuous and observed frequently as somatic mutations without correlation with amyloidosis or were encoded by other germline genes. The replacement of Met at position 4 is expected to improve stability of AL324, indicating a significantly destabilizing mutation at one or more other positions. One candidate is the replacement of Thr by Asn at position 74. Although this would nominally be considered an innocuous replacement, exchanging one small, neutral amino acid for another, it has been found that introduction of either asparagine or aspartic acid into a beta-strand is destabilizing [18] with the impact of aspartic acid sufficient to completely overcome the free energy of folding for the typical variable light chain. Asn74 has been reported in three kappa light chains, two from amyloidosis patients [17], [19] and one from a light chain deposition disease patient [20].

AL700 also appears to be structurally challenged. Gly at position 16 adds significant flexibility to an important beta turn and will be destabilizing or impair the ability of the protein to fold. Replacement of Arg24 by Trp would be expected to decrease the solubility of the protein and destabilize it. Trp 24 has been documented in one kappa light chain, a cryoglobulin [21].The replacement of Tyr86 by Phe, often considered an exchange of similar amino acids is very significant in this case. Tyr86 forms a so-called tyrosine corner [22]. In this case, it forms a buried hydrogen bond to the carbonyl of Asp82, which itself forms a conserved salt-bridge with Arg61. So124 replaces Ser31 with Val. Although position 31 is highly variable among light chain, introduction of Val at this position has been seen in only two other kappa proteins. One formed amyloid [23] while the other was associated with light chain deposition disease [24]. Comparable amino acids replacements of consequence are not readily apparent in AL292 although several positions are missing from the sequence.

### Tissue distribution of AL κ1b amyloid

**Figure 2:**
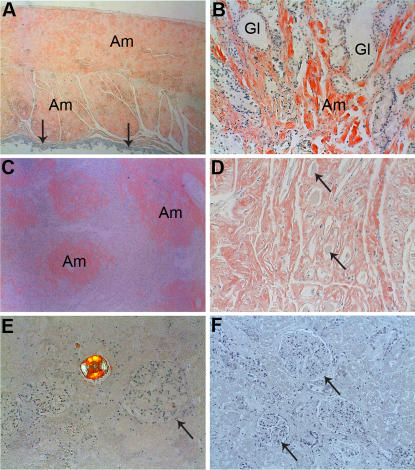
Demonstration of the variation in deposition pattern in the different organs. A. Cross-section of small intestine of AL312. The muscularis propria is converted into amyloid masses (Am). The mucosa is indicated by arrows. B. Section from the stomach of AL Es305 showing large amounts of amyloid between glands (Gl). C. Section of the spleen of AL90. Amyloid (Am) is seen distributed mainly in follicles. D. Section of the tongue of AL Es305 showing large amounts of amyloid. Residual striated muscle fibers (arrows) are seen. E. Kidney section of AL90. The glomeruli (arrow) are virtually free of amyloid. There is a tubular amyloid cast in the middle of the figure. F. Kidney section of AL Es305. The glomeruli (arrows) are free of amyloid.

The distribution of amyloid deposits in the four AL κ1b patients is given in Table 1. Since this is a retrospective study, not all organs were available from the patients. Although all four individuals died from their amyloid disease rather than from multiple myeloma, it is obvious that the degree of amyloid infiltration varied highly, not only between organs but also between patients. Patient 366 had a very pronounced systemic amyloidosis with severe involvement of many parts of the body including both parenchymatous and non-parenchymatous organs. This is the only AL κ1b patient with notable deposits in the liver. Most remarkable was the very pronounced gastrointestinal deposits in patients 312 and 90 (Fig. 2A). These were the main cause of death in patient 312. Also patient 366 had fairly large amount of gastrointestinal amyloid (Fig. 2B) but more as part of the severe systemic disease. Analysis of specific tissue structures may be of particular interest concerning a possibility of tissue affinity for individual light chains. Here can be noted that the intestinal tunica muscularis propria was severely affected in patients 312, 366 and 90 and moderately affected in case Es305 while tunica muscularis mucosae was completely spared in patients Es305 and 312, although topographically closely situated and of similar cell constitution. In patients 366 and 90 on the other hand, both layers contained amyloid.

**Table 1:** Distribution of amyloid in available Congo-red stained sections from the eight studied individuals with systemic AL amyloidosis and in who the amyloid proteins were derived from the κI gene O18-O8 or the κIII gene L2-L16.

Case (pI*)	Es305 (5.36)	312 (4.68)	366 (4.09)	90 (4.4)	700 (9.8)	So124 (9.7)	292 (N.D)	324 (N.D)
Lung								
Alveoli		+	++++	+++	-	+	+	+
Vessels		+	+++	+++	+	+	+	++
Heart								
Myocardium	-	-	+++	+++	+++	+++	++	++
Vessels	+	-	+++	++	++	+++	++	+++
Epicardium	-	+	+++				++	+++
Tongue	++++		+++		+	++		+++
Liver								
Parenchyma	-	-	++	-	++++	++++	++++	++++
Vessels	+	-	+++	+	++	+++	++	++
Pancreas								
Parenchyma		-	++	++	+++		+	
Vessels		+	+++	++			++	
Spleen								
Parenchyma		-	++	+++	++++	++++	++++	++++
Vessels		-	+++	+++	++	+++	+++	
Kidney								
Glomeruli	-**	-	+	-**	+++	++++	++++	+
Vessels	-	+	++	(+)	++	+++	++	++
Stomach								
Mucosa		-	+	+			++	
M.mucosae		-	-	++			-	
Submucosa		-	++	++			+	
M.propria		++	++	++			-	
Ileum								
Mucosa	-	-	++	+	+++	+++	+++	++
M.mucosae	-	-	+++	++	+++	+++		+++
Submucosa	++	+	+++	++	+++	++	+	+++
M.propria	++	++++	+++	+++	-	+	-	++
Skin	++	-			+			
Dermis	+	?		++				
Subcutis								
Adrenal cortex	-		+		++++	+++		
Thyroid	+	+		++++	++	++++	++	++
Pituitary		-						+

Empty space, no tissue available; -, no amyloid; +, one or a few small amyloid deposits; ++, moderate amount of amyloid at several locations; +++, pronounced amyloid deposits; ++++, more than 50% of the tissue replaced by amyloid.

* pI for the variable part of the light chain.

** Amyloid cast found in some tubular lumina.

Only the two patients 366 and 90 had splenic amyloid, in both cases with sago distribution (Fig. 2C). The lack of amyloid in the heart, except for minimal deposits in some vessels, in two of the cases is remarkable. The two other individuals had pronounced and diffuse myocardial deposits. Tongue amyloid was particularly pronounced in patient Es305 (Fig. 2D).

### Tissue distribution of AL κ1b compared to AL κ3a amyloid

As seen from Table 1 there are conspicuous interindividual variations in tissue distribution in both AL subgroups. Yet, there are some striking differences between the two AL subgroups. Pronounced parenchymal liver amyloidosis with hepatomegaly was seen in all κ3a patients but in none of the κ1b individuals. Also, glomerular involvement differed strongly between the two subgroups. The complete lack of glomerular deposits in 3 of the κ1b patients contrasts against the pronounced infiltration in 3 of the κ3a cases (Fig. 2E and 2F). There were also tendencies to be other deposition pattern differences between the two groups, e.g. in the lung and in intestinal involvement. As seen in Table 1, the patients with light chains derived from the κ3a germline have similar deposition pattern demonstrated as a severe systemic amyloidosis affecting most organs investigated.

## Discussion

While much effort has been performed in order to understand amyloidogenesis in general and fibrillogenesis in AL-amyloidosis, very little has been done concerning factors important in tissue localization of deposits. The remarkable variation in distribution of amyloid deposits in systemic AL-amyloidosis has been noted in many studies [6], [25]–[29]. Differences in appearance and prognosis between amyloidosis of AL κ and AL λ have sometimes been suggested [16], [30]. However, studies performed on association of immunoglobulin light chain subgroups with organ manifestations have mainly been based on clinical symptoms and no detailed analyses of tissue involvement associated with specific light chain gene products have been undertaken.

Occurrence of AL-amyloidosis is a consequence of a clonal expansion of immunoglobulin light chain producing plasma cells and a production of an amyloidogenic light chain. A very high variability between different AL-proteins is obvious and finding structural elements linked to tissue distribution of amyloid is therefore extremely difficult. The present study is an effort to partially overcome this problem. Four individuals with terminal systemic AL-amyloidosis were studied and in whom the fibril protein was of κIb type. Thus, the protein sequences were as similar as can be obtained from different AL-proteins. However, several differences between the four AL-proteins were evident and these may influence both fibrillogenicity and tissue targeting.

One of the main aims of the present study was to analyze whether AL-proteins from different individuals, where the monoclonal protein originated from the same V_L_ gene showed any obvious resemblance in amyloid deposition pattern. From Table 1 it is clear that the distribution of amyloid deposits varied highly between the four patients in spite of the O18-O8 gene origin of the light chains and the great sequence similarities. The most striking and remarkable similarity was the lack of significant renal involvement in any of the cases. It can be noted from Table 1 that three out of four individuals with AL-protein of L2-L16 gene origin had pronounced renal deposits, including glomeruli. The calculated isoelectric points of the variable part of the four kappa-I proteins were highly different from those of the two kappa-III chains for which calculation was possible. Glomerular basement membranes are rich in heparan sulfate proteoglycans and therefore negatively charged. Light chains with high pI may therefore be more easily trapped in glomeruli. This putative effect of isoelectric points of the immunoglobulin light chains has to be studied further. Presently, there is nothing known concerning a possible effect of a single amino acid substitution on tissue targeting. Although our study does not directly point to this possibility, such effects may exist. The amino acid sequence probably affects the deposition pattern since the light chains of the κ3a group are deposited in a more similar manner.

Glycosylation is among the identified risk factors for AL-amyloidosis [10] and may also be important for determining in which organs amyloid deposits occur. It has been suggested from an experimental rat study that glycosylated immunoglobulin light chains preferably deposit in the liver [31]. This was not verified in the present study in which both glycosylated and nonglycosylated κ3a proteins formed heavy liver deposition.

A third possible important variable in the distribution of amyloid, in addition to amino acid substitutions and glycosylation, is the protein cleavage pattern and presence of constant region peptides. Discussion of single amino acid substitutions and their effects on properties such as isoelectric point of the variable domain is relevant only if it is the full-length sequence that determines the amyloidogenic properties of a light chain. Otherwise the very complex cleavage patterns have to be taken into consideration. Most AL-proteins are C-terminally truncated and lack a large piece of the constant region. They are most often ragged, particularly C-terminally but also to some part N-terminally. It is still unknown whether truncation takes place before or after fibril formation but cleavage has not been ruled out as an important event in fibrillogenesis. The finding in this (data not shown) and some earlier studies [32], [33] have shown that in addition to the main N-terminal fragment of a monoclonal light chain, AL-proteins often contain small fragments of the constant region. This might indicate that cleavage comes after fibril formation. Occurrence of full-length light chains in all deposits is in accordance with this assumption although the possibility that deposits entrap light chains from the circulation has not been ruled out. However, the situation may be more complex since AL-proteins, mainly containing the constant region have been described [3]–[5].

AL amyloid is unique among the spectrum of amyloid diseases in that it develops from the products of approximately 50 light chain variable domain germline genes. As such, it might be considered to represent some 50 different diseases, each with its own inherent capability of preferential targeting of one or more organs or tissues. However, as demonstrated in this report, patient-specific variations of deposition are found in detailed pathological examination even when the amyloidogenic light chain is derived from a single germline gene. This property likely originates from somatic mutations that occur naturally in light chains during the course of immune system diversification. Although variability originating from patient factors cannot be excluded, much less heterogeneity of organ deposition is found in other systemic amyloidoses, which usually originate from a single gene. We propose that each light chain exhibits one or more determinants of organ-specificity, which originate from primary structure and post-translational modifications. Eventual identification of such determinants could lead to improved treatment of patients with AL amyloidosis, and has the potential to contribute to the development of organ-specific drug delivery systems relevant to many diseases.

## Materials and Methods

### Patients

Tissue material from the eight individuals, who all died from systemic AL-amyloidosis of κ-type, was available in the tissue bank of the laboratory. The use of this material for protein studies has been approved by the ethical committee of Uppsala University Hospital.

The nature of the material made it impossible to obtain consent from the individuals or even relatives since some specimens are 30 years old. We therefore got permission (paragraph 257, Ups 01-083) from the Ethical Committee at Uppsala University Hospital to use this historical material for histological and protein studies without individual consent.

### Purification of amyloid fibril proteins

Amyloid-rich tissues (intestine in patient 312, pericardium in patient 366, liver in patient 292, tongue in patient Es305, thyroid in patient 90 and spleen in patients 700, 324 and So124) were stored at −20°C after the autopsy. Amyloid fibrils were extracted by repeated homogenizations as described [34].The protein sequences of AL 366, AL Es 305, AL 700 and AL So124 have been described previously [17], [23], [35], [36]. Fibrils from AL 312, AL 292 and AL 90 were dissolved in 6 M guanidine HCl-Tris HCl buffer, pH 8.0, containing 0.1 M dithiothreitol and the major fibril protein was purified by repeated gel filtrations as described [37], [38]. Pooled fractions were dialyzed against distilled water and lyophilized.

### Electrophoretic methods

Fibrils were dissolved in sample buffer containing 4% sodium dodecylsulfate (SDS) and subjected to SDS-PAGE which was performed as described [39].

### Structural studies of AL 312

SDS-PAGE was run to evaluate the amyloid fibril protein fractions [40]. Samples of the AL protein, as well as of pure tryptic peptides, were hydrolyzed and analyzed by an automatic amino acid analyzer (Applied Biosystems 421 A, Foster City, CA). Automatic Edman degradation was performed using two different types of sequencers (Applied Biosystems model 477A and Hewlett Packard, model 241). The carboxymethylated amyloid fibril protein was digested with trypsin and endoproteinase Asp-N [36]. Peptides were purified by reverse phase high performance liquid chromatography (RP-HPLC) as described [36]. Tryptic peptides were analyzed by the matrix assisted laser desorption/ionization time-of-flight mass spectrometers (MALDI TOF) Voyager-DE STR (Applied Biosystems) and Ettan MALDI TOF (Amersham Biosciences) and by the hybrid instruments quadrupole/ TOF LC MS/MS; QSTAR (Applied Biosystems) and Q-Tof2 (Micromass UK Limited, Manchester, UK) and the ion trap instrument LCQ DECA (Finnigan ThermoQuest, London, UK). The companies kindly performed all mass analyses as one of several test samples.

### Structural studies of AL 90

Automatic Edman degradation of the purified protein was performed for 47 cycles. The protein was digested with trypsin and the resulting peptides were purified and sequenced by Edman degradation as for AL protein 312.

### Structural studies of AL 292 and AL 324

The extracted light chain proteins 292 and 324 were analyzed by SDS-PAGE and visualized by Coomassie Brilliant Blue. Appropriate bands were excised and treated for in-gel digestion as described [41]. Briefly, the dye was extracted and the gel pieces were equilibrated with ammonium bicarbonate, then dried, and a solution of porcine modified trypsin was allowed to soak into the gel. After overnight incubation, the generated peptides were analyzed by Peptide Mass Fingerprinting on an Ultraflex MALDI TOF/TOF instrument from Bruker, Bremen, Germany. The instruments settings were optimized for analytes up to 4000 Da and the matrix was alfa-cyano 4 –hydroxy cinnamic acid. To improve amino acid sequencing using Post Source Decay, the digest was N-terminally sulfonated using the CAF-MALDI Sequencing kit from GE Healthcare, Uppsala, Sweden [42]. Peptides were then fragmented in the LIFT mode. Sequences were manually interpreted. Fibril material of AL 292 was also dissolved in 6 M guanidine HCl as described [3] and separated on a Sepharose 6B-CL gel filtration column. Pooled fractions were further analyzed by Edman degradation for 14 cycles and thereby the first 14 amino acid residues of AL 292 could be confirmed.

## Supporting Information

Text S1 (0.03 MB DOC)
