# Supplementary material for: Germ Line Origin and Somatic Mutations Determine the Target Tissues in Systemic AL-Amyloidosis
Source: PLoS One. 2007 Oct 3;2(10):e981. doi: 10.1371/journal.pone.0000981 (PMC1989140; doi:10.1371/journal.pone.0000981)
Supplement: Text S1 — (0.03 MB DOC) [file pone.0000981.s001.doc]

**Supporting Information**

##### Results

*Analyses of AL 312*

The elution pattern of the amyloid fibril protein from the Sepharose 6B CL column revealed a small void volume peak and a retarded double peak with shoulders on both sides. Selected fractions from the largest peak were rerun on a Sephacryl S 300 column which resulted in a rather symmetrical peak. SDS-PAGE of the protein material showed bands corresponding to molecular masses of about 5, 8 and 11 kDa. Also, there was a band corresponding to the full-length κ light chain.

N-terminal analysis of the main fraction gave the amino acid sequence of positions 1 to 46 (Fig.1). About 10% of the material yielded a sequence corresponding to positions 109 to 130. Nine cycles of Edman degradation of a more retarded peak material from the Sepharose gel filtration revealed, however, four polypeptides in a ratio of about 1: 1: 1: 1 and the sequences could be interpreted to positions 1 to 9, 46 to 54, 109 to 117 and 127 to 135 (Fig.1). Edman degradation of the protein and of purified peptides derived after digestions with trypsin and endoproteinase Asp-N, resulted in the AL protein sequence, except for positions 143-145. This tryptic peptide was, however, established from the mass analyses.

A verification of the complete AL protein sequence was established by mass analyses of the tryptic peptides on two different MALDI TOF instruments and from sequence data obtained from the Q-TOF TM 2, the Q-STAR and from the ion trap LCQ DECA instruments. The mass analyses revealed that some of the serine-residues at specific positions were modified by the addition of a mass of 28, which could correspond to a formylation. We have no explanation for this modification.

The amyloid fibril protein was rather fragmented, but the largest of the fragments was found to be composed of 206 residues. The heterogeneity in size was disclosed by N-terminal analyses and by SDS-PAGE of the different size exclusion chromatography fractions (data not shown) The smallest polypeptide fraction detected was about 6 kDa. The N-terminal analyses revealed N-termini at seven different positions within the variable as well as the constant region.

The amino acid sequence of the AL protein shows that the light chain is derived from κ1b germline gene and with a J-segment of JκII. Nine of the positions within this germline gene are mutated, namely positions 13, 30, 36, 49, 70, 76, 89, 93 and 95. The amino acid sequence is very similar to those of AL 90, AL 366 and Es 305 (Fig 1).

### Analyses of AL 90

SDS-PAGE of amyloid fibrils, revealed at least three predominating protein bands at 9-13 kDa. This 9-13 kDa complex was purified and used for the structural analyzes of the AL-protein.

Within the determined region, 5 mutations were found in positions 16, 31, 32, 72 and 76. AL 90 has a J-segment of JκII type.

N-terminal analysis of the protein fraction revealed the amino acid sequence of residues 1 to 48. Tryptic peptides gave the sequence of peptides covering positions 1-18, 19-39, 43-45, 46-61, 62-76, 77-101, 98-103, 62-103, 104-107, 109-126, 109-128, 146-149 and 184-188. In addition, fragments starting at positions 104 and 146 were identified. No overlapping peptides for positions 61-62, 103-104 and 107-109 were obtained.
